# Exercise induced hypercoagulability, increased von Willebrand factor and decreased thyroid hormone concentrations in sled dogs

**DOI:** 10.1186/1751-0147-56-11

**Published:** 2014-02-07

**Authors:** Anne KH Krogh, Pernille Legind, Mads Kjelgaard-Hansen, Louise Bochsen, Annemarie T Kristensen

**Affiliations:** 1 Department of Veterinary Clinical and Animal Sciences, University of Copenhagen, Groennegaardsvej 3 Street, Frederiksberg CDK-1870, Denmark

**Keywords:** Canine, Exercise, Gastric lesions, Hemostasis, Sled dogs, Thromboelastography, Thyroid hormones

## Abstract

**Background:**

Sled dogs performing endurance races have been reported to have a high incidence of gastric erosions or ulcerations and an increased risk of gastro intestinal bleeding leading to death in some cases. In addition, these dogs also become hypothyroid during training and exercise. Canine hypothyroidism has been shown to correlate with decreased von Willebrand factor antigen and potentially increased bleeding tendency. Whether increased gastro intestinal bleeding risk is exacerbated due to changes in the hemostatic balance is unknown. The aim of this study was to investigate the hemostatic balance in sled dogs before and after exercise and in addition evaluate any correlation to thyroid status. Twenty sled dogs have been assessed in untrained and trained condition and immediately after exercise. The first sample was collected in the autumn following a resting period, and subsequently the dogs were exposed to increased intensity of training. After four months the peak of physical condition was reached and a 68 km long sled pulling exercise was performed. Samples were collected before and immediately after the exercise. Evaluated parameters were: plasma thromboelastographic (TEG) R, SP, α and MA, activated partial thromboplastin time (aPTT), prothrombin time (PT), fibrinogen, von Willebrand factor (vWf), D-dimer, platelet number, thyroid hormones, hematocrit and C-reactive protein (CRP).

**Results:**

Exercise induced an overall hypercoagulable state characterized by significant decreases of TEG R and SP and an increase of α, increased concentrations of plasma vWf and decreased aPTT. In addition, a proinflammatory status was seen by a significant increase of serum CRP concentrations. Thyroid status was confirmed to be hypothyroid as training and exercise induced significant decrease of thyroxin (T4), free thyroxin (fT4) and thyroxin stimulating hormone (TSH) concentrations. Fibrinogen decreased significantly and PT increased. The training-induced changes showed correlation between T4, fT4 and aPTT and correlation between TSH and fibrinogen. Exercise-induced changes showed correlation between T4 and PT.

**Conclusions:**

Exercise was associated with a hypercoagulable state and an increase of vWf concentration in this group of sled dogs. Decreased thyroid hormone concentrations after training and exercise were confirmed, but were associated with increased and not decreased vWf in this group of sled dogs.

## Background

Humans, horses and sled dogs have increased risk of gastric lesions and ulcerations due to exercise [1-3]. The cause of the lesions is thought to be combinations of gastric hyperacidity and mucosal breakdown and up to 49% of dogs performing strenuous exercise have ulcerations, erosions, gastric hemorrhage or a combination of these [1]. In a canine study evaluating death after strenuous exercise 13% of the dogs died due to blood loss attributable to gastric ulceration [4]. Whether the increased gastro intestinal bleeding risk in dogs is exacerbated due to changes in the hemostatic balance is unknown and the pathophysiology for increased GI bleeding is not fully apparent, but the hemostatic system is a potentially important factor. Hemostasis normally maintains a proper balance between coagulation and fibrinolysis which is essential for prevention of bleeding or thrombus formation. The hemostatic system is influenced by several factors, and in humans it is described how physical training and strenuous exercise can shift the hemostatic balance towards hypercoagulation [5], for example due to microtrauma [6] and not towards hypocoagulation. A limited number of studies have evaluated exercise-induced changes in hemostasis in dogs. One study included beagle dogs performing submaximal exercise in 16 minutes which revealed no significant hemostatic changes [7]. Another study included sled dogs performing sled-pulling activity for 6.4 km [8], and indicated platelet activation by a significant decrease in mean platelet component (MPC) [8]. However, it is currently unknown whether moderate exercise or training affects the hemostatic properties of sled dogs in a way similar to humans. The hemostatic balance in humans is also affected by thyroid hormone concentrations. Humans with hyperthyroidism are procoagulant as evidenced by increased factor VIII, IX, von Willebrand factor (vWf), fibrinogen, and plasminogen activator inhibitor 1 (PAI-1) which can lead to increased thrombus formation [9], while humans with hypothyroidism are hypocoagulant due to decreased FVIII, vWf and fibrinogen which can lead to increased bleeding tendency [10-12]. Decreased vWf antigen has been demonstrated in dogs with hypothyroidism [13], and in sled dogs decreased concentrations of thyroid hormone concentrations following training and exercise have also been reported [14-16].

The aim of this study was to evaluate the hemostatic balance in sled dogs before and after exercise, to evaluate changes in thyroid hormones and subsequently evaluate the possible correlation between the induced changes. Increasing our knowledge on the hemostatic capacity of racing sled dogs is important due to their increased risk of gastric hemorrhage.

## Methods

The study was a prospective longitudinal study including 20 intact Scandinavian husky sled dogs (11 males and 9 females) with a median age of 5 years (range 1 to 10 years) and a 26.1 kg median body weight of males (range 23–31 kg) and 18.4 kg of females (range 17–20 kg). The dogs were housed in the same outdoor facilities (Jämtlands län, Sweden) and received identical feeding and training. The feeding was adjusted during the study period of increasing training intensity, in order to maintain a constant total body condition score of 4–5 out of 9 [17]. The dogs were all determined to be healthy based on physical examination as well as hematologic and serum biochemistry results. Several dogs tested positive by fecal flotation for helminthes at the first examination and received anthelminthic treatment (pyrantel embonate, 15 mg/kg). The dogs received no other medication one month before the beginning of the study or during the study period. The study was approved by the Ethic Committee for Animal Welfare in Sweden and by the Departmental Ethics and Administrative Committee at the University of Copenhagen, Denmark.

### Sample collection

All dogs were sampled three times. The first sample (untrained) was collected early in October before the training season started, while the second sample (trained 4 months) was collected 4 months later in February. In the interim period the training intensity was gradually increased until the animals achieved peak of physical condition. The dogs had not been active 24 hours before the sampling, and were fasted 15 hours before sampling. The third sample (trained 4 months + 68 km run) was collected 24 hours after the trained sample, and within 30 minutes after the 68 km long sled pulling activity. The pulling activity was performed in paths of medium difficulty with a mean speed of 13.9 km/h, and the dogs pulled a mean weight of 17.5 kg each. The dogs were fed during the exercise. All sample sets were collected at the same time (between 1 pm and 4 pm).

Blood was collected by jugular venipuncture, using minimum stasis and a 21-gauge butterfly needle. Blood samples were collected into one serum tube (4 mL, Vacuette), two citrated tubes (2.7 mL, BD Vacutainer) and one EDTA plastic tube (2 mL, BD Vacutainer), in that order. The citrate and EDTA tubes were inverted carefully five times immediately after sampling to ensure mixing of blood with the 3.2% trisodium citrate or EDTA. Serum and EDTA blood samples were subsequently used to measure the biochemical, thyroid profiles and CBC to assess the health and hormone status of the animals. Serum tubes for measuring thyroid hormone concentrations and 3.2% citrate tubes for coagulation profile or TEG were centrifuged at 4000 × g for 120 seconds and the serum and plasma were separated and collected within 30 minutes after sampling. Plasma and serum samples were immediately placed on dry ice and transferred to -80°C within 48 hours. EDTA tubes were stored at 5–8°C until hematological analysis which was performed within 48 hours. Blood smears were made within 30 minutes after sampling. Other analyses were performed within 14 days after sampling.

Hematology analyses were performed using an ADVIA 2120i (Siemens), and biochemistry analyses including C-reactive protein (CRP) were performed using an automated clinical chemistry analyzer (ADVIA 1800 Chemistry System, Siemens) following the manufacturer’s instructions. An Immulite2000 (Siemens) was used to measure serum thyroxine (T4), non-protein bound serum thyroxin (fT4) and serum thyrotropin (TSH) by the use of solid-phase enzyme-labeled chemiluminescent competitive immunoassays (Siemens) for T4 and fT4 and solid-phase enzyme labeled chemiluminescent immunometric assay (Siemens) for TSH.

The following coagulation parameters were measured using an automated coagulometric analyzer (ACL Top500, Instrumentation Laboratory): activated partial thromboplastin time (aPTT), prothrombin time (PT), von Willebrand factor (vWf) and fibrinogen. Plasma D-Dimer was measured using an immunometric flow-through principle (D-Dimer Single Test, NycoCard READER II, Medinor A/S) according to manufacturer’s instructions. Pooled plasma samples from five healthy dogs with known values, confirmed through serial measurements, were used as internal quality control material for the plasma based coagulation assays.

TEG analyses were performed on citrated plasma samples as previously described [18], using a computerized thromboelastograph (TEG 5000 Hemostasis Analyzer System, Haemonetics) with continuous data acquisition. In brief, samples were thawed in a water bath at 37°C, and activated using a solution of recombinant human tissue factor (TF) (Innovin, Dade Behring) at a final TF dilution of 1:50,000 [18]. The parameters chosen for further evaluation were R (reaction time), split point (SP), angle (α) and maximum amplitude (MA). The TEG analyses were run for at least 60 minutes.

All assays were calibrated and controlled according to the manufacturers’ recommendations.

The results were analyzed using GraphPad Prism version 4.01 for Windows (GraphPad Software). To evaluate if a change in hemostatic capacity or thyroid hormones was induced by training and/or exercise, the differences in parameters across the states were calculated (i.e. trained 4 months compared to untrained; and trained 4 months + 68 km run compared to trained 4 months). If differences were normally distributed, a student’s *t*-test testing deviation of mean from zero was performed. If a distribution was not normally distributed, a Wilcoxon signed rank test was performed to test if median was different from zero. To evaluate if there was a correlation between the changes in hemostatic parameters and the changes in thyroid hormone concentrations, a spearman correlation analysis was performed. If a correlation was demonstrated, a linear regression analysis was implemented. The significance level was set to *P* < 0.05 in all tests.

## Results

The median of values, mean of difference and standard deviations of the clotting times, fibrinogen, vWf and the four TEG parameters (R, SP, α and MA), platelet counts and CRP as well as serum thyroid hormone concentrations, for all three sample sets are summarized in Table 1.

**Table 1:** Median values, 25% and 75% percentiles, mean of difference and standard deviation (SD) of concentrations of the thyroid gland hormones: thyroxin (T4), free thyroxin (fT4) and thyroid stimulating hormone (TSH), the secondary hemostatic markers: activated partial thrombin time (aPTT), prothrombin time (PT), fibrinogen, von Willebrand factor (vWf), d-dimer and thromboelastography parameters: reaction time (R), split point (SP), angle (α) and maximal amplitude (MA), platelet numbers (Plt) and C-reactive protein (CRP) measured in 20 sled dogs when they were untrained, trained for 4 months and trained for 4 months and had performed a 68 km run

	**Untrained**				**Trained**				**Trained**	
					**4 months**				**4 months**	
									**+ 68 km run**	
	**Median**	**25%; 75%**	**Mean of diff ± SD**	**Median [25%; 75%]†**	**Median**	**25%; 75%**	**Mean of diff ± SD**	**Median [25%; 75%]†**	**Median**	**25%; 75%**
		**(percentiles)**		**(percentiles)**		**(percentiles)**		**(percentiles)**		**(percentiles)**
T4 (nmol/L)	**22.7**	18.5;29.3	-5.46 ± 8.2**		**17.4**	13.7;21.0	-6.11 ± 5.1***		**8.7**	6.8;15.6
fT4 (pmol/L)	**13.8**	12.2;17.1	-1.88 ± 3.2*		**12.6**	10.5;14.6	-2.05 ± 2.4**		**9.5**	7.9;13.1
TSH (ng/mL)	**0.16**	0.13;0.26	-0.06 ± 0.1**		**0.11**	0.08;0.16		-0.02 [-0.07;0.01]**	**0.07**	0.04;0.11
aPTT (sec)	**10.3**	10.1;10.7	-0.5 ± 0.5**		**9.85**	9.6;10.2	0.02 ± 0.2		**9.95**	9.4;10.3
PT (sec)	**6.5**	6.2;6.8	0.19 ± 0.3**		**6.7**	6.4;6.9		0.3 [0.2;0.5]***	**6.95**	6.7;7.4
fibrinogen (g/L)	**2.74**	2.4;3.4		-0.2 [-0.7;-0.06]**	**2.48**	2.2;2.7	-0.17 ± 0.2**		**2.32**	2.2;2.4
vWf (%)	**104.0**	93;116	0.7 ± 17.8		**98.3**	87;116.9	20.26 ± 14.0***		**124.5**	110.9;135.1
d-dimer (mg/L)	**0.1**	0.1;0.2			**0.1**	0.1;0.1				
R (min)	**3.95**	3.5;4.6	0.31 ± 1.48		**4.14**	3.6;5.5		-1.1 [-1.9;-0.8]***	**3.05**	2.8;3.3
SP (min)	**3.35**	3.0;3.8	0.47 ± 1.28		**3.7**	3.2;4.7		-0.9 [-1.4;-0.65]***	**2.8**	2.5;3.1
α (degree)	**58.4**	42.6;65.2	1.61 ± 16.7		**59.1**	40.3;66.4	12.6 ± 12.0**		**70.1**	65.6;73.8
MA (mm)	**24.75**	21.4;28.0	-3.87 ± 3.9**		**20.00**	18.6;23.2	-1.8 ± 2.2**		**18.85**	17.1;20.9
Plt (10^9^/L)	**424**	338;456	-25.6 ± 87.4		**375**	315;438	2.4 ± 67.8		**354**	316;428
CRP (mg/L)	**2.0**	0.9;9.75	-2.2 ± 16.63		**3.35**	0.85;8.7	11.03 ± 9.09***		**16.35**	8.1;27.9

*P <0.05, **P <0.01, ***P <0.001. All values are determined by use of *t*-test except †values, which are determined by use Wilcoxon signed-rank test.

Exercise (trained 4 months + 68 km run) induced significant changes in R, SP, α, MA, vWf, PT, fibrinogen and CRP. A significant change in concentration of thyroid hormones T4, fT4 and TSH was observed to be induced both by training for 4 months and after exercise. Training for 4 months also induced statistical significant changes of aPTT, PT, fibrinogen and MA. D-dimer could not be measured after exercise in several samples (12/20) due to lipemia, and this parameter was omitted from further analysis. No changes in platelet concentration were observed. Hematocrit was measured and was not correlated to the other parameters.

A significant correlation between training-induced changes in T4 and aPTT (r = 0.53, *P* =0.02) and fT4 and aPTT (r = 0.52, *P* =0.02) were observed, as well as between TSH and fibrinogen (r = -0.61, *P* =0.005). Correlation between exercise-induced changes in T4 and PT (r = -0.49, *P* =0.03) was also observed.

**Figure 1:**
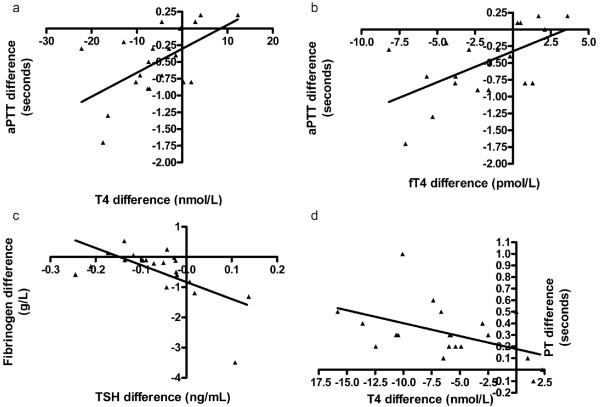
**Associations between thyroid hormones and hemostatic parameters. (a)** Training-induced linear association between thyroxin (T4) concentration and activated partial thromboplastin time (aPTT) in 20 sled dogs (r^2^ =0.33, *P* =0.008). **(b)** Training-induced linear association between free thryroxin (fT4) concentration and activated partial thrombin time (aPTT) in 20 sled dogs (r^2^ =0.34, *P* =0.007). **(c)** Training-induced linear association between thyroxin stimulating hormone (TSH) and fibrinogen concentrations in 20 sled dogs (r^2^ =0.39, *P* =0.003). **(d)** Exercise-induced linear association between thyroxin (T4) concentration and prothrombin time (PT) in 20 sled dogs (r^2^ =0.22, *P* =0.035).

The subsequent linear regression revealed significant linear correlation between training-induced changes of T4, fT4 and aPTT, and between TSH and fibrinogen and also between exercise-induced changes of T4 and PT Figure 1 (a-d).

## Discussion

In this study, exercise (training 4 months + 68 km run) was associated with a procoagulant state characterized by TEG shortened R and SP time and increased α angle as well as significantly decreased aPTT and increased vWf. Training and exercise also induced decreased fibrinogen and MA. These findings are similar to humans where the influence of exercise on hemostatic parameters lead to an increase in procoagulant activity based on steeper angle and faster clot formation evaluated by ROTEM [19] and decrease in aPTT [5]. Although in humans an increase in platelet number and platelet morphology indices were reported [20]. Further an increase in the physiologic inhibitors of coagulation: protein S, protein C, antithrombin, and increase in plasma concentration of d-dimer were also reported [5].

Since the main hemostatic finding was increased procoagulant activity, hemorrhage caused by systemically compromised hemostasis as part of the pathophysiology of the GI bleeding risk, reported in sled dogs, is less likely [1,4,21]. On the other hand prolonged hypercoagulable states associated with long distance racing activity could make dogs vulnerable to thrombosis. However, the procoagulant activity may be balanced by an increase in fibrinolytic activity as seen in humans [5], and could be interesting to pursue in future studies of sled dogs.

The induction of increased procoagulant activity could be speculated to be caused by platelets activated by absorbed intestinal endotoxin [22], as increased intestinal permeability has been demonstrated in sled dogs [21,23] or by the proinflammatory state documented by the increased CRP after the 68 km sled pull. Other procoagulant activating mechanisms could include neutrophil activation, which in turn activates platelets and vice versa [24]. Inflammatory foci could be the result of micro-thrombi formed in the capillaries of skeletal muscles [6] or inflammatory foci in the GI tract due to ischemic injury [25].

In humans shortened a PTT is most often seen as a preanalytical error caused by in-vitro activation of coagulation due to inappropriate sampling, handling or storage. The preanalytical errors in this study are assessed to be minimal, due to a highly standardized protocol. Several conditions such as cancer, myocardial infarction, hyperthyroid disorders, diabetes, and pregnancy have been correlated with shortening of aPTT and increased aPTT-associated clotting factors in humans leading to increased tendency to thromboembolic events [26]. In the present study the sled dogs had an exercise-induced shortening of the aPTT and an increase of vWf and the dogs had decreased concentrations of thyroid hormones as opposed to humans with hyperthyroidism. Whether an acute phase reaction could correlate to the shortening of aPTT as in humans [27] was investigated here by looking for a correlation between CRP and aPTT, but no correlation was documented.

Training and exercise was associated with significantly decreased concentrations of thyroid hormones which was consistent with previous studies [14-16]. Furthermore, a correlation between thyroid hormone concentration and shortened aPTT was documented. Thyroid hormones are influenced by several mechanisms related to maintaining energy balance in the body [28]. Exercise can affect the balance of human thyroid hormones and changes induced by different types, severity and duration of exercise have shown conflicting results [29,30]. The same conflicting results have been reported in dogs where short time exercise leads to increased thyroid hormone concentrations [31,32], whereas beagles performing long time tread mill exercise [33] and sled dogs performing endurance exercise or training have shown significantly decreased serum thyroid hormone concentrations [14-16]. The sled dogs in these previous studies performed either prolonged endurance exercise or training. In the present study, the dogs were evaluated in an untrained state, trained state and after moderate exercise, as opposed to a prolonged endurance exercise. We demonstrate a moderate decrease in serum concentration of T4 and fT4 in training and a more profound decrease after the 68 km exercise. The exercise in this study was not as strenuous as previously described, but a similar decrease in concentration of T4 and fT4 as in strenuous exercise was seen [15]. Variables that could account for the decreases are multiple and could include exercise-induced alterations in distribution or metabolism of hormones [34], altered protein binding of thyroid hormones due to exposure to cold temperatures [35], changed melatonin production which is related to light and exercise [36,37] and diurnal fluctuation [38]. The contribution of the latter is considered to be minimal in this study, due to the similar collection times. The dogs in this study, showed a decrease in T4 and fT4 making it unlikely that a decreased protein binding had a substantial impact on results of the study, since this would lead to an increase of fT4. Lower plasma protein concentrations of competing dogs are seen during long-distance sled races and an increased plasma volume might result in dilution of plasma constituents [39,40]. The T4 and fT4 results were not associated with clinical hypothyroidism because the dogs were evaluated to be clinically healthy.

Overall the study shows that training and moderate exercise decrease serum concentration of thyroid hormones in sled dogs.

The thyroid hormones influence several systems in the body, including the hemostatic system where they modify the balance between coagulation and fibrinolysis [41]. Several changed parameters in coagulation and fibrinolysis related to thyroid dysfunction are described in humans. Hypothyroidism will most often lead to hypocoagulation [41,42] related to a decreased number of platelets due to increased turnover time and inhibition of megakaryopoesis and acquired decreased concentration of vWf-ag [11]. In subclinical hypothyroidism, hypercoagulation is caused by increased plasma concentrations of fibrinogen, vWf, factor VII, factor X and plasminogen activator inhibitor-1 [11,41]. Some of these changes were present in the current study whereas others could not be substantiated. Possible correlation between training-induced or exercise-induced changes in thyroid hormones and changes in the hemostatic capability was investigated, but could not be substantiated for the TEG parameters. Explanations to the statistical significant correlation observed between training-induced changes of T4, fT4 and aPTT and between TSH and fibrinogen, and also between exercise-induced T4 and PT can be multiple, but could be explained by the decreased concentration of thyroid hormones affecting the synthesis and action of coagulation factors. This needs to be further investigated.

## Conclusions

A hypercoagulable state was demonstrated by exercise-induced faster TEG clot formation indicated by decreased R, SP and increased α, and increased vWf concentration. Decreased concentrations of thyroid hormones were confirmed after training and exercise. No correlation between changes in thyroid hormones and global hemostatic parameters were seen.

## Abbreviations

α: Angle; aPTT: Activated partial thromboplastin time; CRP: C-reactive protein; fT4: Free thyroxin; MA: Maximal amplitude; PAI-1: Plasminogen activator inhibitor 1; PT: Prothrombin time; R: Reaction time; SP: Split point; T4: Thyroxin; TEG: Thromboelastography; TF: Tissue factor; TSH: Thyroxin stimulating hormone; vWf: Von Willebrand factor.

## Competing interests

The authors declare no competing interests.

## Authors’ contributions

AK conceived the study, participated in the design and coordination of the study, carried out the hematological, biochemical and hormone studies, performed the statistical analysis and drafted the manuscript. PL participated in the design and coordination of the study, carried out the sample collection and performed statistical analysis. MKH participated in the design of the study, performed statistical analysis, helped to draft the manuscript. LB carried out the hemostatic studies. ATK participated in the design of the study and helped to draft the manuscript. All authors have read and approved the final manuscript.
